# Association of Lifestyle Factors With Blood Pressure Control Among Hypertensive U.S. Adults: An Analysis of the National Ambulatory Medical Care Survey (NAMCS) 2010–2015

**DOI:** 10.7759/cureus.105112

**Published:** 2026-03-12

**Authors:** Moses C Odoeke, Afi T Djaba, Adedoyin Olawoye, Taiwo G Adekanmbi, Hillary C Ugwu, O. B Bomide, Kosisochi E Achara

**Affiliations:** 1 Internal Medicine, University of Toledo, Toledo, USA; 2 Hospital Medicine, King Edward VII Memorial Hospital, Paget, BMU; 3 Internal Medicine, Maimonides Medical Center, New York, USA; 4 Family Medicine, True North Health, Winnipeg, CAN; 5 Intensive Care Unit, Prince Mutaib Bin Abdulaziz Hospital, Sakaka, SAU; 6 Public Health, Purdue University Global, West Lafayette, USA; 7 Public Health, Emory University, Atlanta, USA

**Keywords:** blood pressure control, body mass index, hypertension, lifestyle factors, namcs, outpatient care

## Abstract

Background

Hypertension remains a leading contributor to cardiovascular morbidity in the United States, yet blood pressure control in outpatient settings remains suboptimal. Lifestyle factors and sociodemographic characteristics may influence blood pressure control, but their real-world associations in ambulatory care are not fully understood. This study aimed to assess the association between lifestyle factors, including body mass index (BMI), physical activity, and smoking status, as well as sociodemographic characteristics, and blood pressure control among adults with diagnosed hypertension in U.S. outpatient settings.

Methodology

A cross-sectional analysis was conducted using the National Ambulatory Medical Care Survey from 2010 to 2015. Adults with diagnosed hypertension were included. Blood pressure control was defined as systolic blood pressure <140 mmHg and diastolic blood pressure <90 mmHg. Survey-weighted descriptive and multivariable logistic regression analyses were performed to assess associations between lifestyle factors, sociodemographic characteristics, and blood pressure control.

Results

The final sample included 30,655 unweighted visits, representing 626,165,887 weighted visits nationally. Obesity was independently associated with lower odds of blood pressure control compared with normal BMI (odds ratio = 0.857; 95% confidence interval = 0.774-0.949; p = 0.003). Patients with private insurance had higher odds of blood pressure control than those insured through Medicaid. Non-Hispanic Black adults demonstrated significantly lower odds of blood pressure control compared with non-Hispanic White adults. Documented exercise and tobacco counseling were not independently associated with control.

Conclusions

Blood pressure control in U.S. outpatient settings is influenced by BMI, insurance status, and race or ethnicity, highlighting the need for targeted and sustained interventions beyond routine lifestyle counseling.

## Introduction

Hypertension has been one of the most common and modifiable cardiovascular disease risk factors in the United States and has remained the major contributor to morbidity, mortality, and expenditure in healthcare [1]. Regardless of the abundance of effective antihypertensive drugs, a significant percentage of adults diagnosed with hypertension in the United States have inadequate blood pressure control [2,3]. Uncontrolled blood pressure is a major risk factor associated with increased risk of stroke, myocardial infarction, heart failure, chronic kidney disease, and premature mortality [4]. Therefore, the better management of blood pressure is the primary topic of social concern and one of the priorities of national prevention of the burden of cardiovascular diseases [5].

Lifestyle is a crucial determinant in hypertension development as well as its management. Evidence-based recommendations are always keen on the significance of nonpharmacologic interventions, such as weight control, physical activities, smoking control, and alcohol control in moderation, as elements of hypertension management [6,7]. Being overweight is closely linked to high blood pressure due to its effects on the body, such as increased sympathetic functions, insulin resistance, and vascular dysfunction [8]. Similarly, lack of exercise contributes to reduced cardiovascular fitness and endothelial dysfunction, while tobacco use and excessive alcohol consumption promote vascular inflammation and hypertension [9,10].

Despite the clinical practice guidelines that advocate lifestyle modification as the initial and supplementary method of blood pressure management, compliance with healthy behavior is still very low in the majority of hypertensive adult patients [11]. The adoption and maintenance of healthy lifestyle behaviors may also be influenced by sociodemographic factors such as access to healthcare, as well as by clinical factors such as the presence of comorbid conditions [12,13]. Outpatient clinical environments may limit opportunities for regular lifestyle counseling and follow-up because of time constraints, competing clinical demands, and limited clinical resources [14]. Real-world trends of lifestyle behaviors of hypertensive patients and their relationships with blood pressure control are therefore not necessarily well defined, especially in the ordinary ambulatory care settings [15].

The knowledge of the association between lifestyle and blood pressure management in an outpatient context is needed to inform clinical practice and population health efforts [16]. Conversely, visit-based data available on outpatients provide a great deal of information on the relationship between lifestyle practices and blood pressure outcomes in the existing healthcare delivery systems [17]. These analyses could be used to identify the risk factors that can be modified and could be addressed in the routine care to enhance hypertension management and improve the associated disparities in cardiovascular outcomes [18].

This study used the National Ambulatory Medical Care Survey (NAMCS) for analysis. NAMCS is a nationally representative dataset of outpatient care visits in the United States to physicians and contains in-depth data on patient demographics, diagnoses, vital signs, and selected health behaviors [19]. NAMCS provides a one-of-a-kind chance to study blood pressure management in adults with hypertension in the practice and to determine the correlation between lifestyle factors and the level of treatment results [20,21].

The main objective of the study is to assess the association between lifestyle factors, including body mass index (BMI), physical activity, and smoking status, and blood pressure control among adults diagnosed with hypertension in U.S. outpatient settings. This analysis will inform clinical intervention, preventive counseling, and evidence-based practice to improve the outcomes in managing hypertension and cardiovascular health in the country by identifying lifestyle habits related to effective or ineffective blood pressure control.

## Materials and methods

Study design and data source

This study employed a retrospective cross-sectional design using data from the NAMCS collected between 2010 and 2015 [21]. NAMCS is a nationally representative survey conducted annually by the National Center for Health Statistics that captures visit-level data from non-federally employed, office-based physicians across the United States. The survey utilizes a multistage probability sampling design to collect detailed information on patient demographics, clinical diagnoses, vital signs, prescribed medications, and selected health behaviors. Publicly available NAMCS datasets were pooled across the six-year study period to improve statistical power and ensure nationally representative estimates of outpatient visits among adults with hypertension.

Study population

The study population consisted of adult outpatient visits made by individuals aged 18 years and older with a documented diagnosis of hypertension. Hypertension was identified using the NAMCS condition indicator for hypertension, which reflects physician-documented hypertension recorded during the outpatient visit. Visits with missing blood pressure measurements or age information were excluded from the analysis. The unit of analysis was the outpatient visit rather than the individual patient, consistent with the NAMCS survey design.

After applying inclusion criteria and excluding visits with missing or invalid data for blood pressure measurements, BMI, lifestyle counseling indicators, and covariates, the final analytic sample comprised 30,655 unweighted outpatient visits, representing approximately 626,165,887 weighted visits nationally over the study period.

Study variables and measures

The primary outcome of interest was blood pressure control status, which was determined using systolic and diastolic blood pressure values recorded during each outpatient visit. Blood pressure was classified as controlled when systolic blood pressure was below 140 mmHg and diastolic blood pressure was below 90 mmHg. Visits in which either systolic or diastolic blood pressure met or exceeded these thresholds were categorized as having uncontrolled blood pressure.

The primary exposure variables were selected lifestyle-related factors, including BMI, exercise counseling, and tobacco use or exposure counseling. BMI was obtained directly from the NAMCS dataset and categorized based on standard clinical definitions as normal weight (18.5-24.9 kg/m²), overweight (25.0-29.9 kg/m²), and obese (≥30.0 kg/m²). Physical activity was assessed using a visit-level indicator reflecting whether exercise education or counseling was documented during the encounter, which served as a proxy for clinician-recognized physical activity-related risk. Tobacco-related risk was similarly evaluated using documentation of tobacco use or exposure counseling provided at the visit. Alcohol-related variables were initially considered but were excluded from the analysis due to a high proportion of missing data, exceeding 70% of observations.

Additional covariates captured patient demographic and healthcare access characteristics, including age, sex, and primary expected source of payment as a proxy for insurance coverage. The presence of diabetes mellitus was included as a clinical comorbidity and was identified based on physician-reported diagnoses recorded at the visit.

Statistical analysis

All analyses accounted for the complex survey design of NAMCS by incorporating patient visit weights, strata, and primary sampling units to generate nationally representative estimates. Descriptive statistics were used to summarize patient and visit characteristics by blood pressure control status. Statistical comparisons were performed using survey-adjusted t-tests for continuous variables and F-tests for categorical variables. Survey-weighted logistic regression models were constructed to evaluate the association between lifestyle factors and blood pressure control among hypertensive adults. Adjusted models included sociodemographic characteristics and clinical comorbidities to control for potential confounding. Multicollinearity among independent variables was assessed using variance inflation factors, with all variables demonstrating values ranging from 1.02 to 3.36, indicating no evidence of multicollinearity. Statistical significance was determined using a two-sided p-value of less than 0.05. All analyses were performed using Stata version 18 (StataCorp LLC, College Station, TX, USA).

Missing data

This study employed complete-case analysis due to the NAMCS complex survey design, which limited the use of imputation methods. Variables were assessed for completeness before analysis. Alcohol-related counseling variables exhibited a high proportion of missing data (70.52%) and were excluded from the study to preserve analytic validity and reduce the risk of bias associated with incomplete data.

Ethical considerations

This study utilized de-identified, publicly available data and did not involve direct interaction with human subjects. As such, it was exempt from institutional review board approval in accordance with U.S. federal regulations governing secondary analysis of publicly accessible datasets.

## Results

Table 1 presents the weighted baseline demographic, clinical, and healthcare access characteristics of hypertensive U.S. adults stratified by blood pressure control status using NAMCS data from 2010 to 2015. Comparisons between patients with controlled and uncontrolled blood pressure were conducted using survey-adjusted t-tests for continuous variables and F-tests for categorical variables to account for the complex sampling design.

**Table 1:** Baseline characteristics of hypertensive U.S. adults by blood pressure control status, NAMCS 2010–2015. The values are presented as weighted means ± standard deviations for continuous variables and weighted frequencies with percentages for categorical variables. Blood pressure control was defined as systolic blood pressure <140 mmHg and diastolic blood pressure <90 mmHg. All estimates account for the complex survey design, including patient visit weights, masked strata, and primary sampling units. Statistical comparisons were performed using survey-adjusted t-tests for continuous variables and F-tests for categorical variables. NAMCS = National Ambulatory Medical Care Survey

Characteristic	Blood pressure uncontrolled (N = 205,975,677)	Blood pressure controlled (N= 420,190,210)	F-test/t-test	P-value
Body mass index (kg/m^2^), mean ± SD	31.07 ± 7.35	30.43 ± 6.97	t = 4.83	<0.001
Patient age in years, mean ± SD	63.26 ± 14.63	64.27 ± 13.84	t = -3.56	<0.001
Sex, n (%)			F = 0.59	0.442
Male	95,219,090 (33.2%)	191,256,676 (66.8%)		
Female	110,756,587 (32.6 %)	228,933,534 (67.4%)		
Body mass index (kg/m^2^) category, n (%)			F = 5.88	0.003
Normal	37,584,944 (30.4%)	86,132,129 (69.6%)		
Overweight	66,558,364 (32.2%)	140,259,892 (67.8%)		
Obese	101,832,368 (34.5%)	193,798,190 (65.5%)		
Insurance type, n (%)			F = 7.67	<0.001
Medicaid	21,643,926 (36.7%)	37,397,736 (63.3%)		
Private	111,852,768 (31.7%)	241,592,806 (68.3%)		
Self-pay/Other	72,478,983 (33.9%)	141,199,668 (66.1%)		
Exercise education/counseling, n (%)			F = 0.11	0.745
No	181,148,933 (32.8%)	370,619,744 (67.2%)		
Yes	24,826,743 (33.4%)	49,570,466 (66.6%)		
Tobacco use/Exposure education/counseling, n (%)			F= 0.003	0.96
No	198,532,004 (32.9%)	405,080,093 (67.1%)		
Yes	7,443,672 (33%)	15,110,117 (67%)		
Does the patient now have diabetes, n (%)			F = 0.26	0.611
No	145,074,122 (33.1%)	293,892,541 (66.9%)		
Yes	60,901,555 (32.5%)	126,297,669 (67.5%)		
Race/Ethnicity, n (%)			F = 8.41	<0.001
Non-Hispanic White	142,426,919 (32%)	302,689,198 (68%)		
Non-Hispanic Black	34,245,894 (38.8%)	54,106,048 (61.2%)		
Hispanic	19,930,758 (32.1%)	42,263,309 (67.9%)		
Non-Hispanic Other	9,372,105 (30.7%)	21,131,653 (69.3%)		

Patients with uncontrolled blood pressure had a higher mean BMI compared with those whose blood pressure was controlled (31.07 ± 7.35 kg/m² vs. 30.43 ± 6.97 kg/m², p < 0.001). In contrast, individuals with controlled blood pressure were slightly older on average than those with uncontrolled blood pressure (64.27 ± 13.84 years vs. 63.26 ± 14.63 years, p < 0.001). Sex distribution did not differ significantly by blood pressure control status (p = 0.442); among males, 191,256,676 (66.8%) had controlled blood pressure compared with 95,219,090 (33.2%) with uncontrolled blood pressure, while among females, 228,933,534 (67.4%) were controlled and 110,756,587 (32.6%) were uncontrolled.

Significant differences were observed across BMI categories (p = 0.003). Blood pressure control was highest among individuals with normal BMI, with 86,132,129 (69.6%) having controlled blood pressure, compared with 140,259,892 (67.8%) among those who were overweight and 193,798,190 (65.5%) among those with obesity. Insurance type was also significantly associated with blood pressure control (p < 0.001). Patients with private insurance demonstrated the highest proportion of controlled blood pressure at 241,592,806 (68.3%), compared with 37,397,736 (63.3%) among Medicaid beneficiaries and 141,199,668 (66.1%) among those who were self-pay or had other forms of coverage.

No significant differences in blood pressure control were observed based on receipt of exercise education or counseling (p = 0.745) or tobacco use or exposure counseling (p = 0.960). Among patients who did not receive exercise counseling, 370,619,744 (67.2%) had controlled blood pressure compared with 49,570,466 (66.6%) among those who received counseling. Similarly, blood pressure control was comparable between those without tobacco counseling, 405,080,093 (67.1%), and those who received such counseling, 15,110,117 (67.0%). The presence of diabetes mellitus was not significantly associated with blood pressure control (p = 0.611), with 126,297,669 (67.5%) of patients with diabetes and 293,892,541 (66.9%) of those without diabetes achieving blood pressure control.

Race and ethnicity showed a significant association with blood pressure control (p < 0.001). Non-Hispanic White patients had a relatively high prevalence of controlled blood pressure, 302,689,198 (68.0%). In contrast, non-Hispanic Black patients had the lowest proportion of controlled blood pressure at 54,106,048 (61.2%) and the highest proportion of uncontrolled blood pressure at 34,245,894 (38.8%). Blood pressure control among Hispanic patients was 42,263,309 (67.9%), while individuals categorized as non-Hispanic Other demonstrated the highest proportion of controlled blood pressure at 21,131,653 (69.3%).

**Figure 1:**
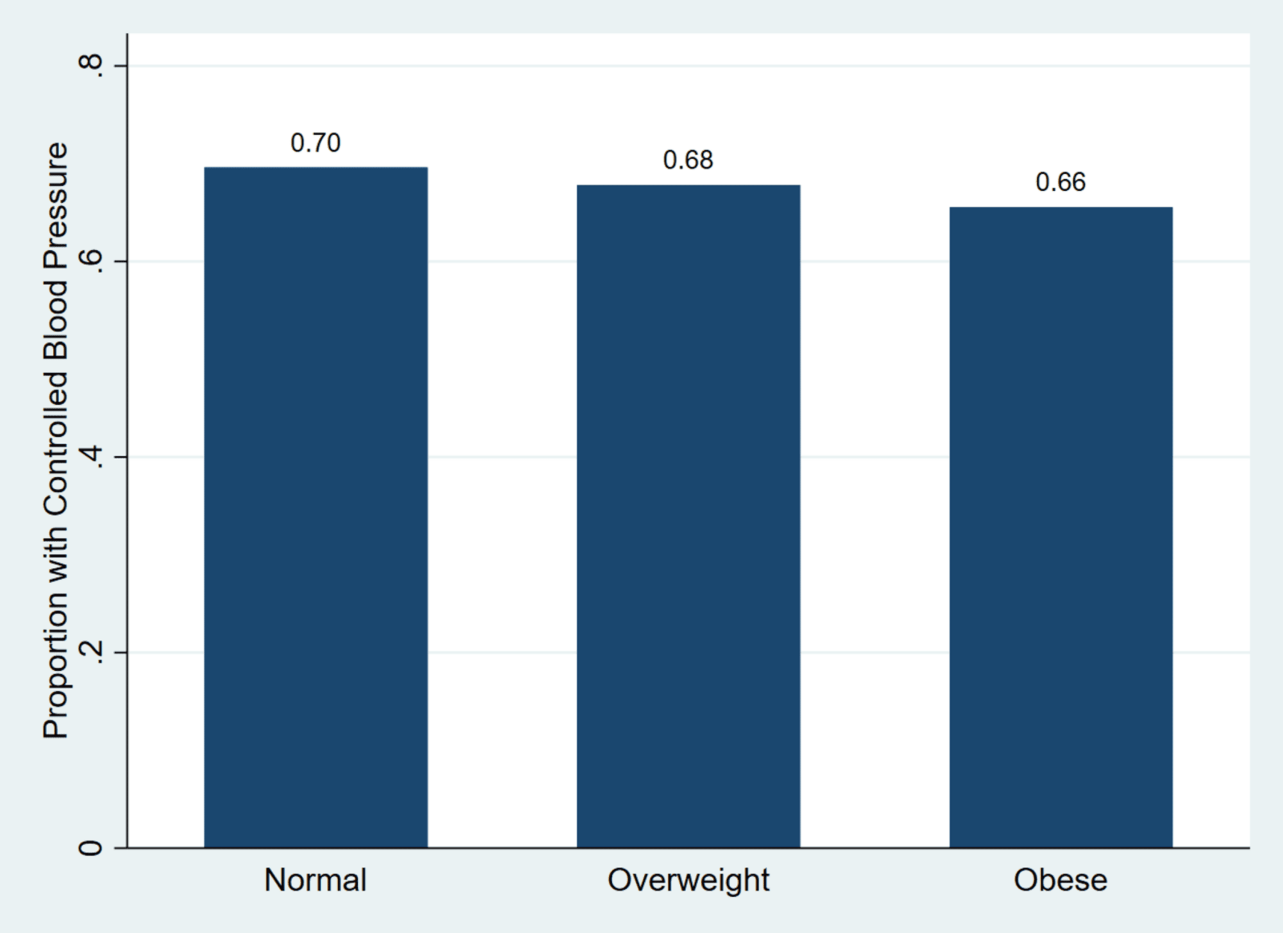
Survey-weighted proportion of hypertensive outpatient visits with controlled blood pressure across body mass index categories, NAMCS 2010–2015. NAMCS = National Ambulatory Medical Care Survey

Figure 1 illustrates the survey-weighted proportion of hypertensive U.S. adults with controlled blood pressure across BMI categories using NAMCS data from 2010 to 2015.

As shown in Figure 1, blood pressure control decreased progressively with increasing BMI. Among patients with normal BMI, approximately 0.70 (70%) had controlled blood pressure, compared with 0.68 (68%) among those who were overweight and 0.66 (66%) among individuals with obesity. This gradient is consistent with the findings presented in Table 1, where a higher proportion of uncontrolled blood pressure was observed among patients with obesity, 101,832,368 (34.5%), compared with those who were overweight, 66,558,364 (32.2%), or had normal BMI, 37,584,944 (30.4%). Together, these results suggest a clear inverse relationship between increasing BMI and blood pressure control in U.S. outpatient settings.

Table 2 presents the results of a survey-weighted multivariable logistic regression examining the independent associations between lifestyle factors, sociodemographic characteristics, and blood pressure control among hypertensive U.S. adults using NAMCS data from 2010 to 2015.

**Table 2:** Multivariable association between lifestyle factors, sociodemographic characteristics, and blood pressure control among hypertensive U.S. adults, NAMCS 2010–2015. This table presents results from a survey-weighted multivariable logistic regression examining factors associated with blood pressure control among adults with diagnosed hypertension in U.S. outpatient settings using data from NAMCS, 2010–2015. Blood pressure control was defined as systolic blood pressure <140 mmHg and diastolic blood pressure <90 mmHg. Male sex, normal body mass index, medicaid, and non-Hispanic White race/ethnicity served as reference categories in the regression analyses. NAMCS = National Ambulatory Medical Care Survey

Variable	Odds ratio	95% confidence interval	P-value
Patient age in years	1.004	1.001–1.007	0.021
Male (vs. Female)	1.044	0.969–1.124	0.259
Body mass index (kg/m^2^) category			
Overweight (vs. normal)	0.932	0.819–1.060	0.283
Obese (vs. normal)	0.857	0.774–0.949	0.003
Exercise education/counseling	0.992	0.859–1.144	0.908
Tobacco use/Exposure education/counseling	1.034	0.847–1.262	0.744
Insurance type			
Private (vs. Medicaid)	1.221	1.075–1.386	0.002
Self-pay/Other (vs. Medicaid)	1.061	0.932–1.209	0.369
Patients with diabetes	1.058	0.968–1.156	0.212
Race/Ethnicity			
Non-Hispanic Black (vs. non-Hispanic White)	0.781	0.690–0.885	<0.001
Hispanic (vs. non-Hispanic White)	1.037	0.895–1.203	0.627
Non-Hispanic Other (vs. non-Hispanic White)	1.050	0.867–1.271	0.618

After adjustment for covariates, increasing age was modestly but significantly associated with higher odds of blood pressure control (odds ratio (OR) = 1.004; 95% confidence interval (CI) = 1.001-1.007; p = 0.021). Sex was not independently associated with blood pressure control, as males had similar odds of controlled blood pressure compared with females (OR = 1.044; 95% CI = 0.969-1.124; p = 0.259). BMI category demonstrated a graded association with blood pressure control. Compared with patients with normal BMI, those with obesity had significantly lower odds of achieving blood pressure control (OR = 0.857; 95% CI = 0.774-0.949; p = 0.003), whereas the association for overweight status did not reach statistical significance (OR = 0.932; 95% CI = 0.819-1.060; p = 0.283).

Neither exercise education nor counseling (OR = 0.992; 95% CI = 0.859-1.144; p = 0.908) nor tobacco use or exposure counseling (OR = 1.034; 95% CI = 0.847-1.262; p = 0.744) was independently associated with blood pressure control in adjusted analyses. Insurance type was significantly associated with blood pressure outcomes. Patients with private insurance had higher odds of controlled blood pressure compared with Medicaid beneficiaries (OR = 1.221; 95% CI = 1.075-1.386; p = 0.002), while no significant difference was observed for those who were self-pay or had other forms of coverage (OR = 1.061; 95% CI = 0.932-1.209; p = 0.369). The presence of diabetes mellitus was not significantly associated with blood pressure control after adjustment (OR = 1.058; 95% CI = 0.968-1.156; p = 0.212).

Race and ethnicity remained independently associated with blood pressure control. Compared with non-Hispanic White patients, non-Hispanic Black patients had significantly lower odds of controlled blood pressure (OR = 0.781; 95% CI = 0.690-0.885; p < 0.001). No statistically significant differences in blood pressure control were observed for Hispanic patients (OR = 1.037; 95% CI = 0.895­-1.203; p = 0.627) or those categorized as non-Hispanic Other (OR = 1.050; 95% CI = 0.867-1.271; p = 0.618) relative to non-Hispanic White patients.

**Figure 2:**
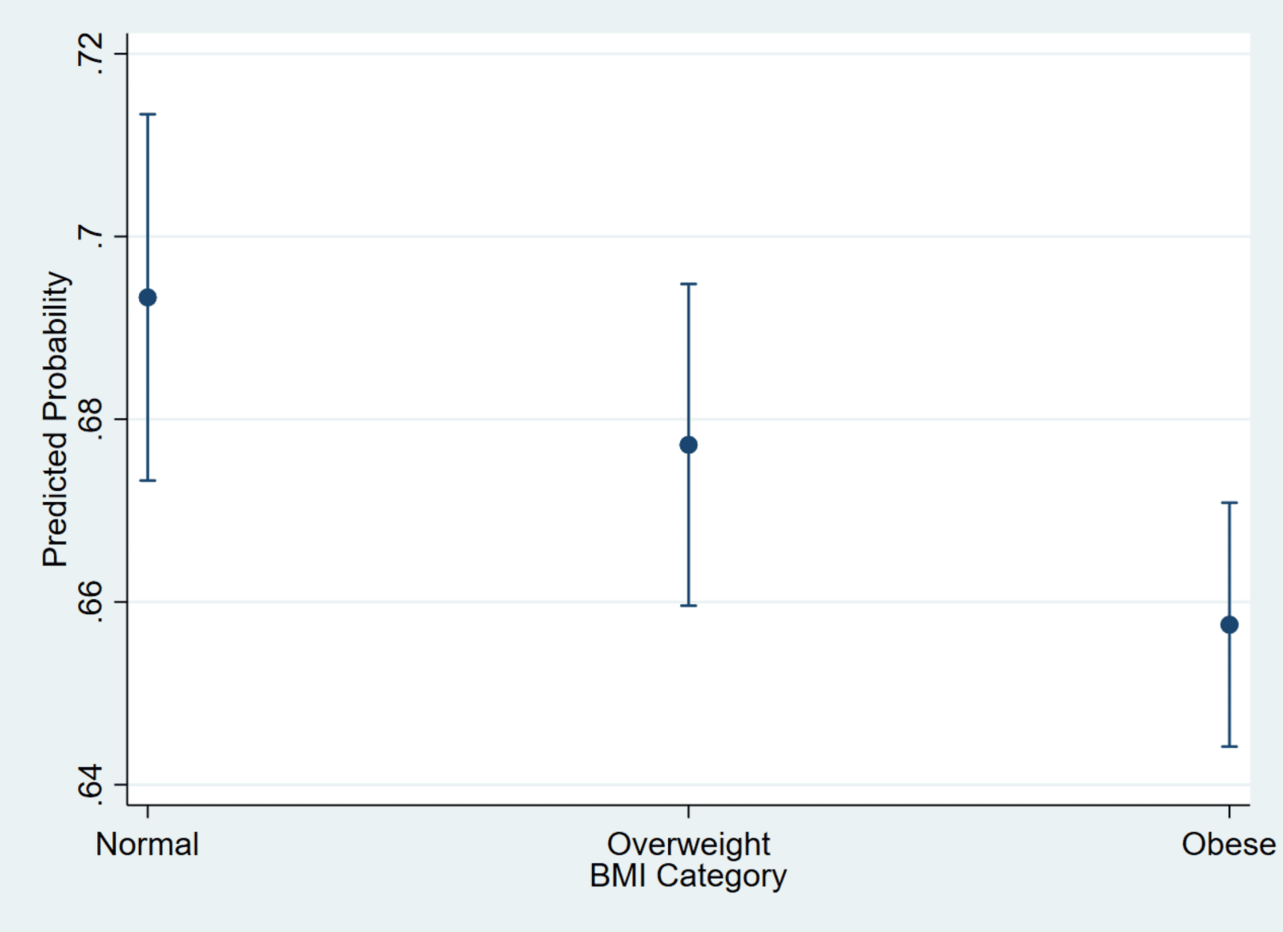
Adjusted probability of blood pressure control by body mass index (BMI).

Figure 2 displays the survey-weighted adjusted predicted probabilities of blood pressure control across BMI categories, estimated from the multivariable logistic regression model presented in Table 2.

Consistent with the adjusted ORs reported in Table 2, the predicted probability of achieving blood pressure control declined with increasing BMI category. Patients with normal BMI demonstrated the highest adjusted probability of controlled blood pressure at approximately 0.69, followed by those who were overweight at approximately 0.68, and those with obesity at approximately 0.66. The separation between BMI categories remained evident after adjustment for age, sex, insurance type, diabetes status, race and ethnicity, and lifestyle counseling variables. Notably, the lower predicted probability among individuals with obesity aligns with the significantly reduced odds of blood pressure control observed for this group in the regression analysis (OR = 0.857; 95% CI = 0.774-0.949), reinforcing the independent association between higher BMI and poorer blood pressure control.

## Discussion

Using nationally representative outpatient data from NAMCS, this study evaluated the association between lifestyle-related factors, sociodemographic characteristics, and blood pressure control among U.S. adults with diagnosed hypertension. Despite longstanding guideline recommendations emphasizing lifestyle modification as a cornerstone of hypertension management [6,7], the findings demonstrate that blood pressure control in routine ambulatory care remains strongly patterned by BMI, insurance status, and race or ethnicity rather than by documented lifestyle counseling alone.

BMI emerged as one of the most consistent correlates of blood pressure control. Both descriptive and adjusted analyses showed a progressive decline in the proportion and probability of controlled blood pressure from normal-weight to obese individuals. These findings are consistent with established evidence linking excess adiposity to hypertension through sympathetic activation, renal sodium retention, insulin resistance, and vascular dysfunction [8]. Importantly, the persistence of this association after multivariable adjustment suggests that obesity remains an independent barrier to achieving blood pressure control in outpatient practice, even among patients already engaged in care.

Older age was associated with slightly higher odds of blood pressure control, a pattern that aligns with prior studies suggesting that older adults may receive more intensive monitoring, pharmacologic treatment, or follow-up compared with younger populations [2,3]. In contrast, sex was not independently associated with blood pressure control after adjustment, indicating that differences in control observed in prior population-level studies may be mediated by other clinical or access-related factors rather than sex alone [1].

Insurance status was a significant determinant of blood pressure control. Patients with private insurance demonstrated better control compared with those insured through Medicaid. This finding supports earlier evidence that healthcare access, continuity, and resource availability influence hypertension outcomes [12,13]. Patients insured through Medicaid may face structural barriers such as limited access to consistent providers or reduced visit continuity, which can hinder optimal chronic disease management.

Marked racial disparities were also observed. Non-Hispanic Black adults had significantly lower odds of blood pressure control compared with non-Hispanic White adults, even after adjustment for insurance, BMI, and comorbid diabetes. This finding mirrors national trends and emphasizes the role of broader structural and social determinants, including healthcare access inequities, chronic stress, and differential treatment responses, in shaping hypertension outcomes [2,4]. The absence of significant differences among Hispanic and non-Hispanic Other groups suggests heterogeneity in risk profiles and care patterns across racial and ethnic populations.

Notably, documented exercise and tobacco-use counseling were not independently associated with blood pressure control. Although lifestyle modification is strongly emphasized in clinical guidelines [6,7], this finding likely reflects the limitations of visit-based counseling documentation in outpatient settings. Prior studies have highlighted that brief counseling during routine visits may be insufficient to achieve sustained behavioral change, particularly in the absence of structured follow-up or multidisciplinary support [14,15]. These results suggest that counseling alone, as captured in NAMCS, may not adequately translate into improved blood pressure outcomes.

Lifestyle modification is a core element of hypertension management and is strongly endorsed by the American College of Cardiology (ACC) and the American Heart Association (AHA) in the 2017 ACC/AHA Guideline for the Prevention, Detection, Evaluation, and Management of High Blood Pressure in Adults [22]. Evidence indicates that weight loss is associated with an approximate 1 mmHg reduction in systolic blood pressure per kilogram lost, the Dietary Approaches to Stop Hypertension eating pattern reduces systolic blood pressure by about 5 to 8 mmHg in hypertensive adults, sodium reduction to 1,500 to 2,300 mg per day lowers blood pressure, and at least 150 minutes per week of moderate-intensity aerobic activity yields an additional 4 to 9 mmHg reduction; moderation of alcohol intake and smoking cessation further improve cardiovascular risk profiles and support blood pressure management [5-7,22]. Despite these established benefits, nationally representative evidence assessing whether documented lifestyle counseling during ambulatory visits translates into blood pressure control remains limited. Analysis of NAMCS data from 2010 to 2015 showed that although diet, exercise, and tobacco counseling were frequently recorded, they were not independently associated with blood pressure control after adjustment for covariates. In contrast, insurance status and race were significant predictors, with privately insured adults demonstrating higher odds of control than Medicaid-insured adults and non-Hispanic Black adults demonstrating lower odds of control compared with non-Hispanic White adults. These findings suggest that documentation of counseling alone may be insufficient to achieve recommended blood pressure targets and highlight the importance of broader structural and equity-focused approaches to hypertension management, including culturally congruent care, improved access to preventive services, and community-based interventions aimed at high-risk populations.

Strengths and limitations

This study leverages a large, nationally representative outpatient dataset, allowing findings to be generalized to U.S. ambulatory care settings. The use of survey-weighted analyses ensures appropriate national estimates, and the combined use of descriptive, multivariable, and graphical analyses strengthens interpretability.

Several limitations should be acknowledged. The cross-sectional design precludes causal inference. Lifestyle behaviors were inferred from counseling documentation rather than direct measures of behavior or adherence, which may underestimate their true impact. Blood pressure measurements reflect single-visit readings and may not capture long-term control. Additionally, information on antihypertensive medication adherence and treatment intensity was not available.

Future research should prioritize longitudinal studies that evaluate sustained lifestyle interventions, particularly structured weight management programs, and their impact on blood pressure control. Studies should also explore mechanisms underlying insurance- and race-related disparities, including healthcare access, continuity, and social determinants of health.

## Conclusions

In this nationally representative outpatient study, blood pressure control among U.S. adults with hypertension varied significantly by BMI, insurance status, and race or ethnicity. Obesity was independently associated with lower odds of blood pressure control, emphasizing the clinical challenges of managing hypertension in patients with excess weight. Patients with private insurance demonstrated better control compared with those insured through Medicaid, highlighting disparities in access and continuity of care. Lifestyle counseling documented at visits was not independently associated with control, suggesting the need for more sustained, structured interventions to improve hypertension outcomes.
